# Effect of Different Reduction Intramedullary Nails on Spiral Fracture of Middle and Lower Tibia

**DOI:** 10.1155/2022/4716978

**Published:** 2022-03-28

**Authors:** Yongxin Shi, Fuqin Li, Weiguo Liang, Jinshi Liu

**Affiliations:** ^1^Department of Orthopedics, Guangzhou Red Cross Hospital, Medical College, Jinan University, Guangzhou 510220, Guangdong, China; ^2^Department of Orthopedics, Univercity of Chinese Academy of Sciences Shenzhen Hospital (Guangming), Shenzhen, China; ^3^Department of Social Welfare Centre, Univercity of Chinese Academy of Sciences Shenzhen Hospital (Guangming), Shenzhen, China

## Abstract

**Objective:**

To compare the efficacy of different reduction and intramedullary nailing in the treatment of spiral fracture of middle and lower tibia.

**Method:**

A total of 96 patients with spiral fractures of middle and lower tibia treated with intramedullary nails were retrospectively analyzed. The patients were divided into closed functional reduction group, open anatomical reduction group, and closed anatomical reduction group according to different treatment methods. The operation time, intraoperative blood loss, intraoperative fluoroscopy times, fracture healing time, fracture nonunion, wound complications, and healing conditions of the three groups were compared.

**Results:**

The operation time and intraoperative fluoroscopy times of patients in the closed anatomical reduction group were significantly increased compared with those in the closed functional reduction group, while the fracture healing time was significantly reduced. However, patients in the open reduction group had significantly more intraoperative blood loss than those in the closed reduction group. The mean follow-up duration of patients was 15.81 ± 3.25 months. Open anatomical reduction was found to have a higher complication rate during follow-up. Specifically, a total of 3 cases recovered after 2 times of surgical treatment. 6 cases showed a small gap at the fracture end which did not affect the function.

**Conclusion:**

In the treatment of middle and lower spiral fracture of tibia, closed anatomical reduction and intramedullary nail internal fixation have shorter fracture healing time, less blood loss, and fewer complications, which can act as the first surgical choice. However, open reduction and intramedullary nailing have a high complication rate, which is not recommended.

## 1. Introduction

Middle and lower spiral fracture of tibia, a common type of fracture in clinical practice, is mostly caused by high-energy injuries and rotational violence [1, 2], which has a high incidence in young patients [3]. Studies have shown that tibial spiral fractures are often associated with posterior ankle fractures [4] and proximal fibular fractures [5] and in severe cases can lead to posttraumatic osteoarthritis and secondary postoperative displacement [6, 7]. However, the efficacy of traditional methods such as superarticular external fixation and ordinary compression plate is not ideal [8]. At present, the effective treatment is closed reduction and intramedullary nail internal fixation [9–11]. However, because of the large medullary cavity in the middle and lower tibia, anatomical reduction often cannot be performed during closed reduction of the fracture, so fracture healing time is prolonged. Therefore, it is necessary to find new effective treatments. Some scholars have studied the clinical efficacy of limited open medial anatomical plate internal fixation in the treatment of middle and lower spiral fracture of tibia and found that it has good efficacy and few complications [12]. Therefore, 96 cases of middle and lower tibial fractures treated with intramedullary nails were retrospectively analyzed to compare the efficacy of closed functional reduction group, closed reduction and intramedullary nailing, and open reduction and intramedullary nailing, providing clinical data support for the effective treatment of middle and lower spiral fracture of tibia.

## 2. Materials and Methods

### 2.1. General Information

A total of 96 patients with spiral fracture of middle and lower tibia admitted to our hospital from August 2015 to June 2020 were collected. According to the different treatment methods, the patients were divided into the following three groups: closed functional reduction group (*n* = 31): anatomic reduction of the fracture is not achieved and the separation of the fracture ends is less than 2.0 mm; open anatomical reduction group (*n* = 29); and closed anatomical reduction group (*n* = 36). An informed consent form was obtained from all patients, and the study was approved by the Ethics Committee of the University of Chinese Academy of Sciences-Shenzhen Hospital (2021263).

Inclusion criteria were as follows: ① unilateral closed fracture of tibia; X-ray or CT examination and other imaging examinations confirmed the diagnosis of middle and lower spiral fracture of tibia, with or without fibular fractures; ② fresh fracture without fracture-related treatment; ③ normal ambulation before fracture; and ④ surgical procedures were consistent with intramedullary nailing.

Exclusion criteria were as follows: ① patients with combined vascular nerve injury; ② patients with osteoporosis; ③ pathological fracture; ④ patients with fracture line involving articular surface; ⑤ multifragmentary fracture; and ⑥ reported impairments that accelerate fracture healing such as combined traumatic brain injury.

### 2.2. Treatment Methods

After admission, all patients were subjected to immobilization and detumescence treatment, external fixation with plaster, and traction immobilization of the posterior calcaneal tuberosity. Surgical contraindications were excluded according to the patient's comprehensive condition. The surgery was performed approximately 7 days after the patient reached the peak of swelling.

Before the surgery, the patients were given prevention of deep venous thrombosis treatment and guidance of toe movement, quadriceps spontaneous contraction, hip and back lifting, and other painless exercises. 30 minutes before surgery, cephalosporin was applied for infection prophylaxis. General anesthesia with endotracheal intubation or arachnoid anesthesia was applied. With the patients supine on the operating table, a pneumatic tourniquet was tied to the heel of the thigh.

Closed functional reduction group: hip flexion was about 90°, knee flexion was 90°–100°, and median longitudinal anterior incision of patellar ligament was about 4.0 cm in length. The patellar ligament was split longitudinally. The inferior fat pad was pushed to expose the bevel from the anterior edge of tibial plateau to tibial tubercle. Positioning needles were inserted in the middle point of the bevel. After the C-arm fluoroscopy guide wire was in the appropriate position, the incision was opened and the guide wire was inserted. Subsequently, after reduction of the fracture under C-arm fluoroscopy, a guide wire was inserted through the fracture end to the distal tibial medullary cavity. Then, reaming was performed. Intramedullary nailing with an appropriate diameter was inserted along the long axis of the tibia to achieve functional reduction of the fracture. Anatomical reduction was unable to be achieved. There was a certain degree of separation at the end of the fracture, and the separation was less than 2.0 mm. The limb length was restored under C-arm fluoroscopy. After correcting the rotation, 3-4 distal locking screws were inserted. After appropriate compression of the fracture end, another 2 locking screws were inserted, and then the wound was sutured.

Open anatomical reduction group: longitudinal incision (length: 4.0 cm) was performed at the fracture end. After exposing the fracture end, fracture reduction under direct vision was conducted. Reduction forceps were applied to fix fracture ends. The periosteum was protected as much as possible during the operation. There was no need to strip the periosteum of fracture end. Then, intramedullary nailing was implanted as in the closed functional reduction group. Impaction of screw or Kirschner wire was inserted at fracture end to increase stability.

Closed anatomical reduction group: previous surgical procedures were the same as the closed functional reduction group. If anatomical reduction of the fracture was not achieved under fluoroscopy, the intramedullary nails were withdrawn and the guide wire was retained in the medullary cavity to rereduce. The blocking screw technique was adopted. Specifically, a guide wire was first inserted into the medullary cavity on the acute angle side formed by the fracture line at the distal end of the fracture and the guide wire. Then, the intramedullary nails were reimplanted. If the reduction was still poor, the second Kirschner wire on the acute angle side formed by the fracture line at the proximal end of the fracture and the guide wire was implanted as a blocking screw. Until the fracture end was anatomically reduced (separation was less than 1.0 mm), the length of limb was restored under C-arm fluoroscopy. After correcting the rotation, 3-4 distal locking screws were inserted. After appropriate compression of the fracture end, two locking screws were inserted. After that, the wound was sutured.

### 2.3. Postoperative Treatment

Cephalosporin was continued to be applied for the prevention of infection within 24 hours after surgery. Early active, painless ankle joint functional exercise and quadriceps contraction exercise were conducted during postoperative anesthetic resuscitation. On the second postoperative day, active lifting of lower limbs and passive knee training were conducted. And meanwhile, the patients were guided to sit up and practice ambulation on crutches at the start of treatment for prophylaxis of deep vein thrombosis. About 2 weeks after the operation, the stitches were removed after wound healing. The time of partial weight-bearing and full weight-bearing of the limb after operation was determined according to the patient's re-examination. The degree of fracture healing was checked in combination with the result of X-ray, and then the patients were guided to walk with weight-bearing.

### 2.4. Evaluation Indicators

The operation time, intraoperative blood loss, intraoperative fluoroscopy times, and fracture healing time were recorded and compared.

### 2.5. Follow-Up

All patients were followed up. All complications during follow-up were recorded, such as nonunion, wound complications, and the presence of small gaps at the fracture end after healing that did not affect the function.

### 2.6. Statistical Analysis

All data were analyzed using SPSS 22.0 statistical software. Measurement data that were consistent with normal distribution were expressed as mean ± standard deviation (SD), one-way analysis of variance was used for comparison between multiple groups, and *t*-test was used for comparison between two groups. Enumeration data were analyzed by the *χ*^2^ test. *P* > 0.05 was considered statistically significant.

## 3. Results

### 3.1. Comparison of Surgical Indicators among Three Groups

Among 96 patients, 69 males and 27 females were included (age: 20–55 years). The surgery-related indicators of the three groups were recorded. The results revealed that the operation time and fluoroscopy times in the closed anatomical reduction group were significantly longer and much more than those in the closed functional reduction group, while the fracture healing time was reduced. However, the intraoperative blood loss of patients in the open anatomical reduction group was significantly more than that in the closed anatomical reduction group (Table 1).

### 3.2. Comparison of Postoperative Healing among Three Groups

**Figure 1:**
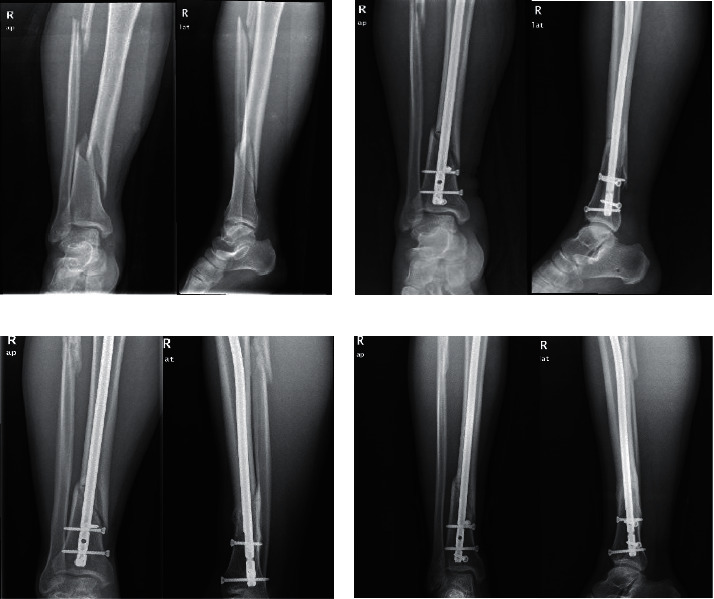
Preoperative and postoperative CT scans of one patient in the closed functional reduction group. Preoperative (a) and postoperative (b) CT scan of the patient; CT scan of the patient at 10 months (c) and 14 months (d) after operation.

CT scan was performed in the three groups to observe the postoperative healing. In the closed functional reduction group, a 28-year-old man fell and caused closed spiral fracture of right lower tibia shaft and upper fibular fracture (Figure 1(a)). Closed reduction and internal fixation were utilized to achieve fracture reduction (fracture displacement <2.0 mm) (Figure 1(b)). Ten months after surgery, a re-examination was performed, and the results of CT showed the fracture line was still in the patient and the fracture was still unhealed (Figure 1(c)). The results of re-examination at 14 months after surgery showed that the fracture had healed and the patient had no pain when walking (Figure 1(d)).

**Figure 2:**
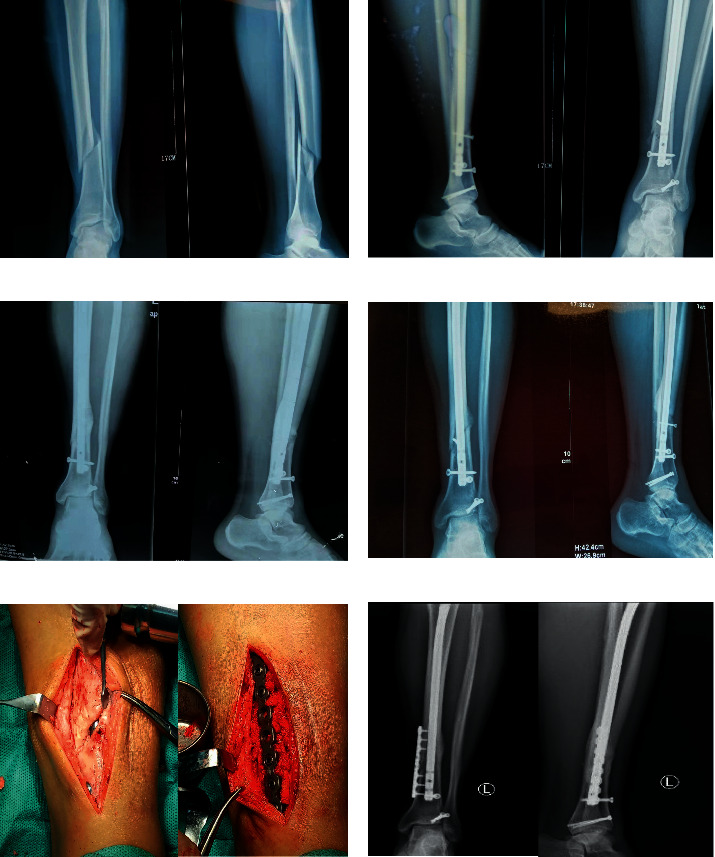
Preoperative and postoperative CT scans of a patient in the open anatomical reduction group. Preoperative (a) and postoperative (b) CT scan of the patient; CT scan of the patient at 18 months (c) and 30 months (d) after operation; (e) the image of the patient who received the second surgery; (f) CT scan of the patient who received the second surgery at 12 months after operation.

In the open anatomical reduction group, a 35-year-old man fell and resulted in spiral fracture of left lower tibia shaft and upper fibular fracture (Figure 2(a)). Open reduction and internal fixation were applied. A poller screw was implanted to increase stability, and the fracture was reduced completely (Figure 2(b)). Re-examination was conducted at 18 months after the operation, and the results showed that the fracture was still not healed (Figure 2(c)). Thirty months after surgery, the fracture was still not healed (Figure 2(d)) and the patient felt pain after walking for more than 100 meters, indicating fracture nonunion. An incision was made to explore, finding large bone defects and inadequate blood supply at the fracture site. Debridement was applied to debride until the fracture site bleeding was restored. Then, the autologous cancellous bone was implanted, and a reconstruction plate was adopted to fix the fracture site and then to increase the stability (Figure 2(e)). Re-examination was conducted at 12 months after the second surgery, and the result showed the fracture was healed (Figure 2(f)).

**Figure 3:**
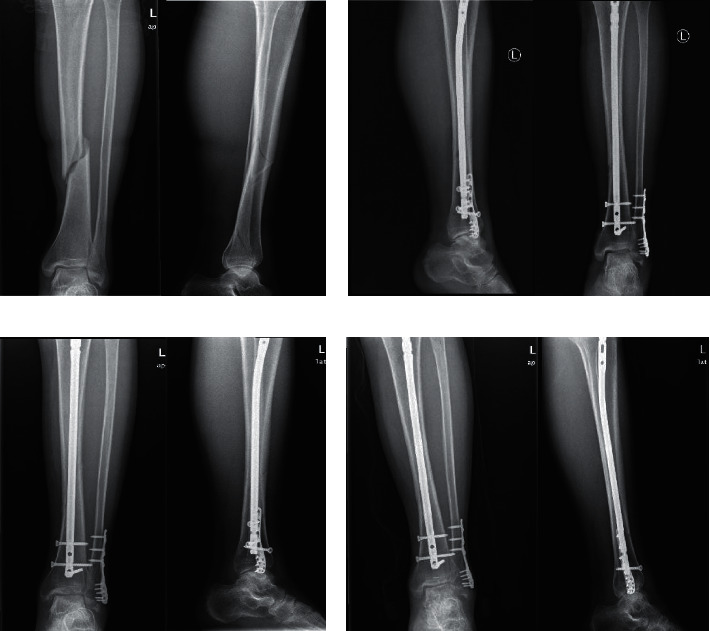
Preoperative and postoperative CT scans of a patient in the closed anatomical reduction group. Preoperative (a) and postoperative (b) CT scan of the patient; CT scan of the patient at 3 months (c) and 5 months (d) after operation.

In the closed anatomical reduction group, a 29-year-old man fell and caused spiral fracture of lower tibia shaft and combined lower fractures of the tibia and fibula (Figure 3(a)). Closed reduction was applied. Specifically, reduction was conducted with poller screws and anatomic reduction of fracture was achieved (fracture displacement <1.0 mm) (Figure 3(b)). The result of re-examination at 3 months after the operation showed that the fracture line disappeared (Figure 3(c)) and the patient could walk with normal weight-bearing (Figure 3(d)). The result of re-examination at 5 months after the operation showed that the fracture line had completely disappeared and the patient could walk normally (Figure 3(d)). All the above results indicated that patients in the closed anatomical reduction group had the shortest postoperative healing time, while patients who received open anatomical reduction still required a second operation.

### 3.3. Comparison of Postoperative Complications among the Three Groups

All patients were followed up. The longest follow-up time was 60 months, and the average time was 15.81 ± 3.25 months. Postoperative complications in the three groups were recorded. The results indicated that, in the open anatomical reduction group, there were 3 patients with wound exudate, including 2 cases of *Staphylococcus epidermidis* infection and 1 case of *Staphylococcus aureus* infection. In the closed anatomical reduction group, there was 1 patient with tibial fracture end blocking the drainage from the screw hole. Three patients in the open anatomical reduction group had unhealed fracture with large gap at the fracture site. The limbs of patients might be affected, and the fracture might reoccur. After the second debridement and bone grafting at the fracture site, the fracture was healed (Table 2). All the above results revealed that the incidence of postoperative complications in the open anatomical reduction group was higher than that in the closed anatomical reduction group.

## 4. Discussion

Middle and lower spiral fracture of tibia, a common type of fracture in clinical practice, is more common in young patients [13]. Intramedullary nails or internal fixation plate was a major method in treatment of middle and lower spiral fracture of tibia [14–16]. The application of internal fixation plate extends healing time, delays the time of ambulation with weight-bearing, and affects blood supply of incision of fracture, thereby causing high fracture nonunion rate, infection rate, and so on [17]. At present, most surgeons prefer intramedullary nailing [18–20]. However, the anatomy of middle and lower spiral fracture site of tibia is special. Specifically, enlargement of the medullary cavity at the distal end of the fracture leads to difficulty in reduction and fixation [21]. Unlike easy achievement of anatomical reduction in oblique fracture or transverse fracture, the spiral fracture can often only achieve functional reduction. Furthermore, the blood supply of the middle and lower tibia is single, so both fracture and surgical procedures are easy to destroy the blood supply of the site of the fracture, thereby resulting in complications such as delayed fracture healing and nonunion [22, 23]. We retrospectively summarized the treatment and healing of the fractures and found that the degree of reduction of the fracture and whether the site of fracture was incised were two key factors affecting fracture healing and efficacy. Good fracture reduction and blood supply protection at the fracture end can achieve perfect healing of middle and lower spiral fracture of tibia. In this study, it was found that all patients in the closed anatomical reduction group achieved good healing with the shortest healing time on average. Also, the patients recovered well without any complications or sequelae. However, in the closed functional reduction group, the patient did not undergo open reduction and the fracture healing time was obviously prolonged. Nevertheless, all 31 patients had good fracture healing without sequelae or serious complications. In the open anatomical reduction group, three patients underwent a second operation and significant complications also occurred in healing patients. Because of less soft tissue coverage and special blood supply in the middle and lower tibia and especially the worst blood supply on the medial surface of the tibia, the periosteum supply becomes the main supplementary source [24]. Also, open reduction, especially the use of reduction forceps, damages the periosteal blood supply [25, 26], thereby causing the occurrence of nonunion.

During this study, it was also found that although the patients had good functional recovery in their limbs, the bone strength was reduced to some extent and the risk of recurrent fracture was increased at a later stage. Besides, the large amount of callus formed at the site of fracture end in patients was different from the callus formed during the healing of the fracture. Healing of fractures relies on internal callus formation [27]. The callus formed in this study belonged to external callus formation and did not participate in the process of creeping substitution, so it had little use for the healing of fractures. Also, the external callus did not appear in large numbers at the fracture line, but at both ends of the fracture line, which suggested that the stimulation to the periosteum might be the cause of internal fixation failure and recurrent fractures.

The findings of closed anatomical reduction in this study are shown as follows: ① anatomical reduction of the tibia was easily achieved in patients without fibula fracture; ② for patients with tibiofibular fracture, preoperative use of calcaneal tuberosity bone traction could help tibial fracture to achieve anatomical reduction during surgery [28]; ③ rational use of muscle relaxants could reduce the difficulty of reduction; and ④ the poller screw technique played a significant role in anatomical reduction of middle and lower spiral fracture of tibia [29–31]. Specifically, after implantation of the intramedullary nail, if the separation of the fracture site was found to be longer than 2.0 mm, the intramedullary nail was pulled out. Then, the Kirschner wire was inserted through the medullary canal at the sharp angle between the distal fracture and the guide wire, and the intramedullary nail was reimplanted. If anatomical reduction was still not achieved, a second Kirschner wire could be inserted and an intramedullary nail was also reimplanted, until the fracture was well reduced.

## 5. Conclusion

To sum up, intramedullary nailing can offer good efficacy for the treatment of middle and lower spiral fracture of tibia. Closed anatomical reduction can act as the first choice of treatment. If anatomical reduction cannot be achieved, closed functional reduction can be applied. However, the use of open anatomical reduction should be reduced due to the high incidence of nonunion.

## Figures and Tables

**Table 1:** Comparison of surgical indicators among three groups.

Group	Operation time (min)	Intraoperative blood loss (ml)	Intraoperative fluoroscopy times (time)	Fracture healing time (week)
Closed functional reduction group (*n* = 31)	101.35 ± 15.36	96.58 ± 19.81	12.20 ± 1.61	38.65 ± 12.36
Open anatomical reduction group (*n* = 29)	98.51 ± 10.33	136.25 ± 10.68^*∗*^	9.31 ± 1.20	39.15 ± 13.49
Closed anatomical reduction group (*n* = 36)	132.58 ± 19.66#	98.28 ± 10.22	23.63 ± 2.99^*∗*^#	12.61 ± 6.39^*∗*^#

F value	8.615	23.25	58.22	45.36
*P* value	0.014	0.019	<0.001	<0.001

The data were expressed as mean ± SD. Compared with the closed functional reduction group, ^*∗*^*p* < 0.05; compared with the open anatomical reduction group, ^#^*P* < 0.05.

**Table 2:** Comparison of surgical complications among the three groups.

Group	Nonunion (case)	Wound complications (case)	Small gap at fracture end (case)	The incidence of complications (%)
Closed functional reduction group (*n* = 31)	0	0	0	0
Open anatomical reduction group (*n* = 29)	3	3	6	41.38 (%)^*∗*^
Closed anatomical reduction group (*n* = 36)	0	1	0	0.028 (%)#

*χ* ^2^ value	—	—	—	92.92
*P* value	—	—	—	<0.001

Compared with the closed functional reduction group, ^*∗*^*p* < 0.05; compared with the open anatomical reduction group, ^#^*P* < 0.05.

## Data Availability

The data used to support the findings of this study are available from the corresponding author upon request.
